# Evolution of the spatiotemporal pattern of China’s grain production in the past 20 years and its driving mechanism

**DOI:** 10.1371/journal.pone.0303258

**Published:** 2024-05-29

**Authors:** Longbo Ma, Binyu Yang, Huijie Zhang, Wenbin Jiang, Liyu Ju

**Affiliations:** 1 School of Economics and Management, Qingdao Agricultural University, Qingdao, Shandong, China; 2 China Food Research & Training Center, Beijing, China; Khwaja Fareed University of Engineering & Information Technology, PAKISTAN

## Abstract

Food security is a goal and means of global sustainable development, and an important component of China’s national security. Based on grain production data from 2000 to 2020, 31 provinces (cities, autonomous regions) in China were used as research units to analyze the spatiotemporal differences and driving forces of grain production in China using a combination of local correlation index, center of gravity transfer model, and geographic detector. The results as follows, ① During the research period, China’s total grain production showed a decrease followed by an increase, and the yield per unit area of grain showed an increasing trend. Corn has become the "largest staple food" in China; ② During the research period, the focus of China’s grain production continued to shift northward, with Heilongjiang, Henan and Shandong provinces becoming the main grain production areas, with Henan being the province where China’s grain production center was located. Among the factors affecting grain yield, the effective irrigation area (0.971) has the strongest explanatory power. Finally, countermeasures and suggestions were proposed from five aspects, stabilizing grain production, reducing grain inventory pressure, implementing regional grain security responsibilities, improving grain circulation efficiency, promoting high-quality grain engineering construction, adjusting grain production structure, strictly implementing farmland protection responsibilities, scientifically applying pesticides, fertilizers and other production materials, emphasizing agricultural infrastructure construction and stabilizing the number of agricultural labor force.

## Introduction

Eliminating hunger and achieving food security are important goals in the Sustainable Development Goals (SDGs). However, affected by global climate change, the Russia-Ukraine conflict, the COVID-19, etc. global food security is facing multiple threats and challenges. As a major food producing country in the world, China, in recent years, had witnessed frequent natural disasters caused by extreme weather, posing a huge challenge to the stability of China’s grain production. At the same time, driven by rapid urbanization and industrialization, China’s grain production center is constantly shifting northward and the grain production and sales pattern has undergone significant changes, especially the high-speed non-agricultural transformation of factors such as farmland and labor, and the issue of "who will grow grain" has attracted widespread attention from all sectors of society. Although the issue of food security in China has shifted from a single issue of quantity security to a combination of quantity security, quality security, consumption security, and ecological security, quantity security remains the foundation of food security. In order to improve China’s food security level, it is urgent to explore and clarify the evolution of China’s food production pattern and its driving factors.

In recent years, the evolution of grain production patterns and their driving forces had attracted the attention of many scholars. Relevant scholars had explored the grain production pattern and its center of gravity transfer process in China using methods such as GAEZ model, center of gravity transfer model [1], ESDA method [2], coefficient of variation [3], spatial autocorrelation, standard deviation ellipse [4] at different scales at the national [5], provincial [6], and county levels [7, 8]. Due to the fact that grain production is a unified process of natural and social production, there are many influencing factors, including labor resource mobility [9], labor aging, feminization [10], human capital stock [11], arable land [12], fertilizer [13], technological progress [14], etc. The factors that have a dominant impact on grain production during different periods are different [15], and can be roughly divided into four categories, including natural factors, capital factors, labor factors, and policy factors [16, 17]. Among them, natural factors include sunshine, temperature, precipitation, natural disasters, etc. Capital factors mainly include technological investment [16], fertilizer application amount and total power of agricultural machinery [18], etc. Labor factors include both the quantity and quality of labor engaged in agriculture [19]. In addition, policy factors are also one of the factors affecting grain production. Fiscal support for agriculture expenditure is one of the influencing factors [20]. If its ultimate impact is considered, policy factors can be examined from the proportion of non-grain crops to the total planting area of crops and the proportion of agricultural output value to total output value [19]. In summary, existing research has provided a solid foundation and reference for the development of this study. However, there are many studies that combine one or two methods for analysis, and there are few studies that combine multiple methods, making it difficult to accurately depict the evolution process of grain production patterns and reveal the impact of complex factor interactions on grain production.

This article is based on grain production data from 31 provinces (cities, autonomous regions) in China(2000–2020), and combined methods are used, including descriptive statistics, local correlation indices, center of gravity transfer models, and geographic detectors, aims to reveal the temporal and spatial characteristics and evolution of grain production in China over the past 20 years, and analyze the important factors and driving mechanisms that affect grain production. In theory, it can compensate for the shortcomings of existing research and enrich the existing research content. To provide scientific basis for stabilizing grain production and ensuring food security, and promote the achievement of sustainable goals.

## Materials and methods

### Research methods

#### (1)Local correlation index

(1)The local correlation index is used to explore the changes in the degree of agglomeration of grain production. The local correlation index Getis-Ord GI* statistic can identify clusters of higher indicator values (hot spots) and clusters of lower indicator values (cold spots), and is commonly used to analyze the degree of aggregation of observations in local space. The formula [21]is as follows.


Gi*(d)=∑j=1nwij(d)xj∑j=1nxj (1)

$G_{i}^{*}(d)$ is standardized, i.e. $Z(G_{i}^{*})=\frac{G_{i}^{*}-E(G_{i}^{*})}{\sqrt{Var(G_{i}^{*})}}$, where $E(G_{i}^{*})$ is the expected value of $G_{i}^{*},Var(G_{i}^{*})$ is Variance of $G_{i}^{*}$, *w*_*ij*_ (*d*) is the spatial weight. If $Z(G_{i}^{*})$ is positive and significant, it indicates that the value of position i and the surrounding area are relatively high (higher than the mean), indicating a high-level clustering area (hot spot area). On the contrary, if the value is negative and significant, it indicates that the value around position i is lower than the mean, and correspondingly shows as a low-level agglomeration area (cold spot area). This article uses this method to analyze the characteristics of changes in the spatial agglomeration pattern of grain production.

#### Center of gravity transfer model

The grain production center of gravity migration model can reflect the spatial distribution and changes of grain production. If a region is composed of multiple secondary regions, the center of gravity coordinates of the i secondary region are (*M*_*i*_, *N*_*i*_), and P is the weight of a certain attribute value in the region. This article uses the center of gravity transfer model to explore the overall motion law of the quantity center of gravity and the production potential center of China’s grain production space. Drawing on the research results of Yu Yuanhe et al [21], the calculation formula is set as follows.


$$\{\begin{array}{c} M=\sum_{i=1}^{n}P_{i}M_{i}/\sum_{i=1}^{n}P_{i}\\ N=\sum_{i=1}^{n}P_{i}N_{i}/\sum_{i=1}^{n}P_{i} \end{array}$$ (2)

In the formula, *M* and *N* are the longitude and latitude coordinates of the grain production center of gravity, respectively. P is the grain yield of the i province (city, autonomous region). *M*_*i*_、*N*_*i*_ is the central longitude and latitude coordinates of the i province (city, autonomous region), calculated using ArcGIS software. This article uses this method to analyze the characteristics of changes in the distribution center of grain production.

#### Geographic detectors

(4)(3)Geographic detectors is used to analyze the driving forces behind the spatiotemporal differences in China’s grain production, including factor detector results analysis and interaction detector results analysis. Geographic detectors are tools for detecting and utilizing spatial differentiation, which can achieve differentiation and factor detection, as well as interaction detection. Among them, differentiation and factor detection are used to detect the spatial differentiation of dependent variable (represented by *Y* below), and to detect the extent to which a certain factor *X* explains the spatial differentiation of attribute *Y*, measured by a q value. The expression is as follows.


$$q=1-\frac{\overset{L}{\underset{h=1}{\sum }}N_{h}\sigma_{h}^{2}}{N\sigma_{h}^{2}}=1‐\frac{SSW}{SST}(SSW_{h=1}^{L}=\sum N_{h}\sigma_{h}^{2},SST=N\sigma^{2})$$ (3)

(3)In the formula, *h* = 1,⋯,*L*, represents the stratification of variable *Y* or factor *X*, i.e. classification or partitioning. *N*_*h*_ and *N* represent the number of units in layer *h* and the entire area, respectively. $\sigma_{h}^{2}$ and *σ*^2^ is the variance of the *Y* values for layer *h* and the entire region respectively. *SSW* and *SST* are the sum of intra layer variances and the total variance of the entire region respectively. The value range of *q* is [0,1], and the larger the value, the more significant the spatial differentiation of *Y*. If the stratification is generated by the independent variable *X*, the larger the *q* value, the stronger the explanatory power of the independent variable *X* on the attribute *Y*, and vice versa.

Then conduct interaction detection. Identify the interactions between different risk factors *X*_*s*_, that is, evaluate whether factors *X*_1_ and *X*_2_ work together to increase or decrease the explanatory power of the dependent variable *Y*, or whether these factors have independent effects on *Y*.The evaluation method is to first calculate the *q* values of two factors *X*_1_ and *X*_2_ for *Y*: *q*(*X*_1_) and *q*(*X*_2_), and then calculate the *q* s of their interaction *q*(*X*_1_∩*X*_2_), and compare *q*(*X*_1_), *q*(*X*_2_) 1, 2, and *q*(*X*_1_∩*X*_2_). The relationship between two factors can be divided into the following categories (Table 1).

**Table 1:** Analysis basis for interaction detection results.

Judgment basis	interaction
*q*(*X*_1_∩*X*_2_)<Min [*q*(*X*_1_), *q*(*X*_2_)]	Nonlinear attenuation
Min [*q*(*X*_1_), *q*(*X*_2_)]< *q*(*X*_1_∩*X*_2_)<Max [*q*(*X*_1_), *q*(*X*_2_)]	Single factor nonlinear attenuation
*q*(*X*_1_∩*X*_2_)> Max [*q*(*X*_1_), *q*(*X*_2_)]	Double factor enhancement
*q*(*X*_1_∩*X*_2_) = *q*(*X*_1_) + *q*(*X*_2_)	independence
*q*(*X*_1_∩*X*_2_)> *q*(*X*_1_) + *q*(*X*_2_)	Nonlinear enhancement

On the basis of existing research, this article starts from four types of influencing factors. Two indicators are selected from natural factors, including grain sowing area and non-disaster area of crops. Three indicators are selected for capital factors, including total power of agricultural machinery, fertilizer application amount, and effective irrigation area. The agricultural labor force quantity is selected as indicator of the labor force factor. Policy factor are examined through financial support for agriculture expenditure. The non-disaster area of crops is obtained by subtracting the disaster area of crops from the total sown area of crops. Before using geographic detectors for analysis, the various influencing factors are first dispersed, and after running the software, the factor detection and interaction detection results are obtained separately. By using interaction detectors, the factors that enhance the explanatory power of the main factors can be identified, thereby determining the main factors affecting grain yield and helping to determine the deep influencing factors affecting grain yield. The interaction detection results show the *q* value after the superposition of two influencing factors, and the larger the value, the stronger the effect.

### Data sources

This study covers 31 provinces (cities, autonomous regions, Access to data in the Hong Kong Special Administrative Region, Macau Special Administrative Region, and Taiwan Province of China is restricted) in China. The data on grain yield, grain planting area, main grain crop yield, and planting area of each province (city, autonomous region) (2000–2020) are mainly sourced from the China Grain Yearbook. The data on the disaster area of crops, total sown area of crops, total power of agricultural machinery, fertilizer application amount, effective irrigation area, agricultural labor force, and financial support for agriculture (2000–2020) are sourced from the China Statistical Yearbook and the China Rural Statistical Yearbook.

## Results

### Analysis of time characteristics of grain production in China

#### Time series changes in total grain production

**Fig 1:**
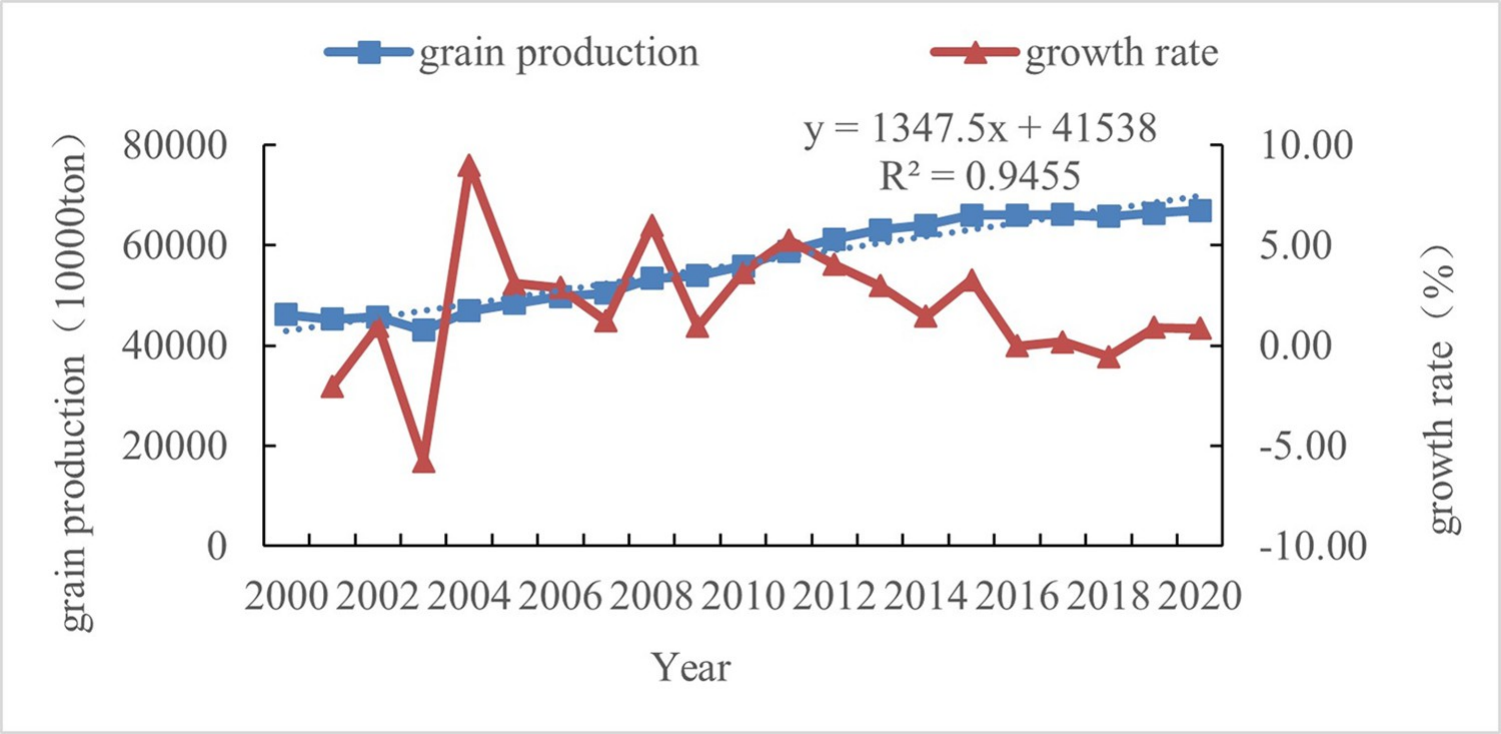
The grain production, growth rate and its trend line equation in China (2000–2020). (Note: The trend line equation takes the time axis as the X-axis and defaults the initial year to 1, the same below).

The overall grain production (2000–2020) showed a trend of first decreasing and then increasing. In 2000, the national grain production was 462.18 million tons, which increased to 669.49 million tons in 2020. During this period, based on the trend of changes in the national total grain production value, it can be divided into two stages, with the period from the year 2000 to 2003 being the first stage. The characteristic of this period is that the national total grain production continued to decline and reached its lowest point (430.7 million tons) in 2003. Since the year 2004, the national total grain production has increased again and returned to the level of total grain production in the year 2000. Since then, the national grain production has fluctuated, the fluctuation is relatively small. The growth rate can also reflect the trend of changes in grain production. In the year 2003, the largest reduction in grain production was 5.77% year-on-year, while in the year 2004, the largest increase in grain production was 9% year-on-year (Fig 1). As for the whole country, the increase in total grain production after the year 2003 was mainly due to various measures such as reducing agricultural taxes, providing direct subsidies for grain, and supporting the construction of grain production zones introduced by the government in the year 2004, which increased the enthusiasm of grain farmers and reversed the trend of decreasing total grain production.

#### Time series changes in grain yield per unit area

**Fig 2:**
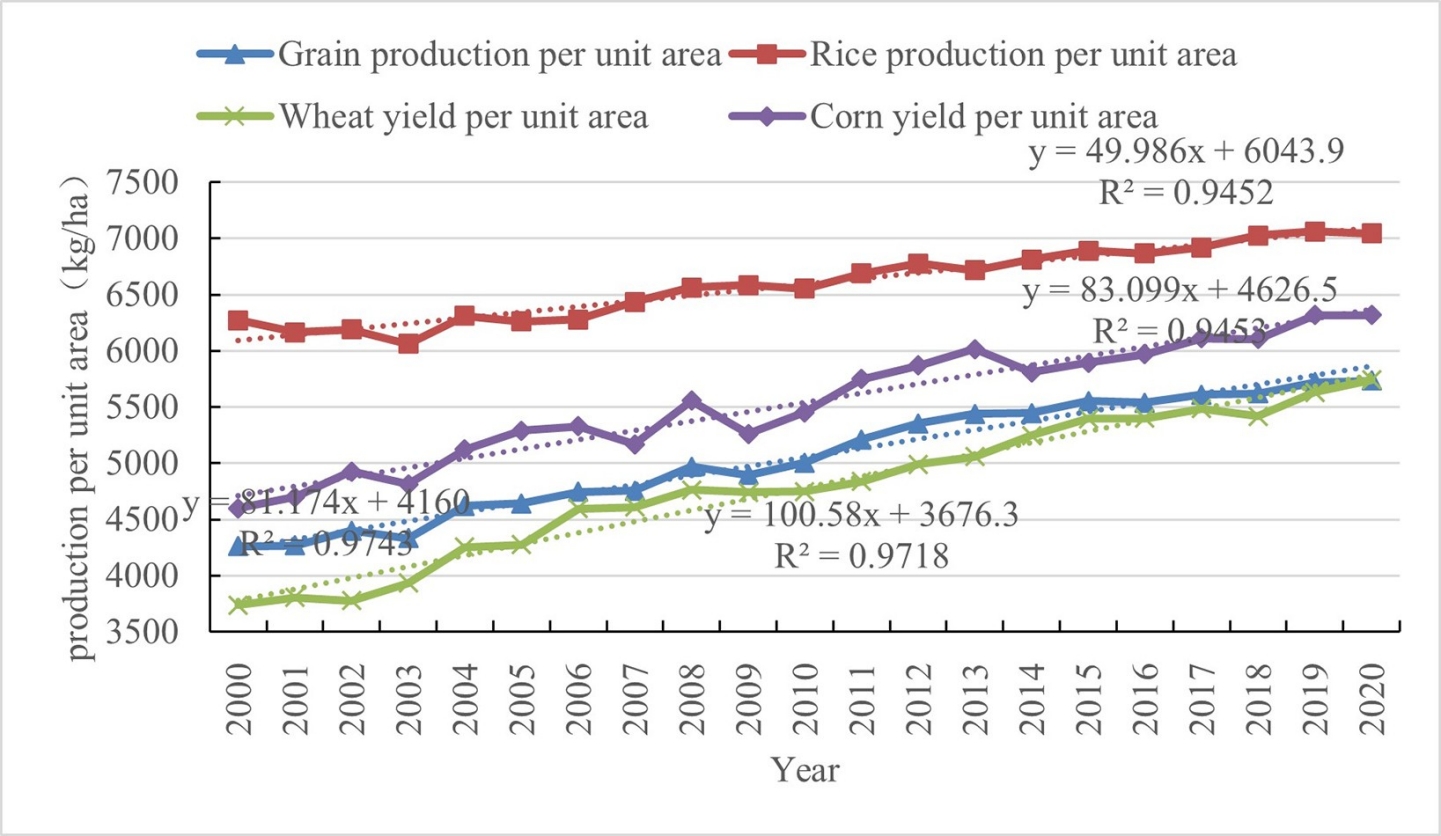
National grain, three major crop unit area yield and their trend line equations (2000–2020).

From the year 2000 to 2020, the national grain yield per unit area increased from 4261kg ha-1 to 5734kg ha-1, showing a stable and increasing trend overall. From the perspective of main crops, the yield per unit area of rice, corn, and wheat increased from 6272 kg ha-1, 4597 kg ha-1, and 3738 kg ha-1 to 7044 kg ha-1, 6317 kg ha-1, and 5742 kg ha-1, respectively (Fig 2), with annual growth rates of 2.07%, 1.52%, and 0.55%. In addition to the impact of the national grain crop planting area, the increase in grain yield per unit area has played an important role in increasing the total amount of grain.

#### Time series changes in grain production structure

**Fig 3:**
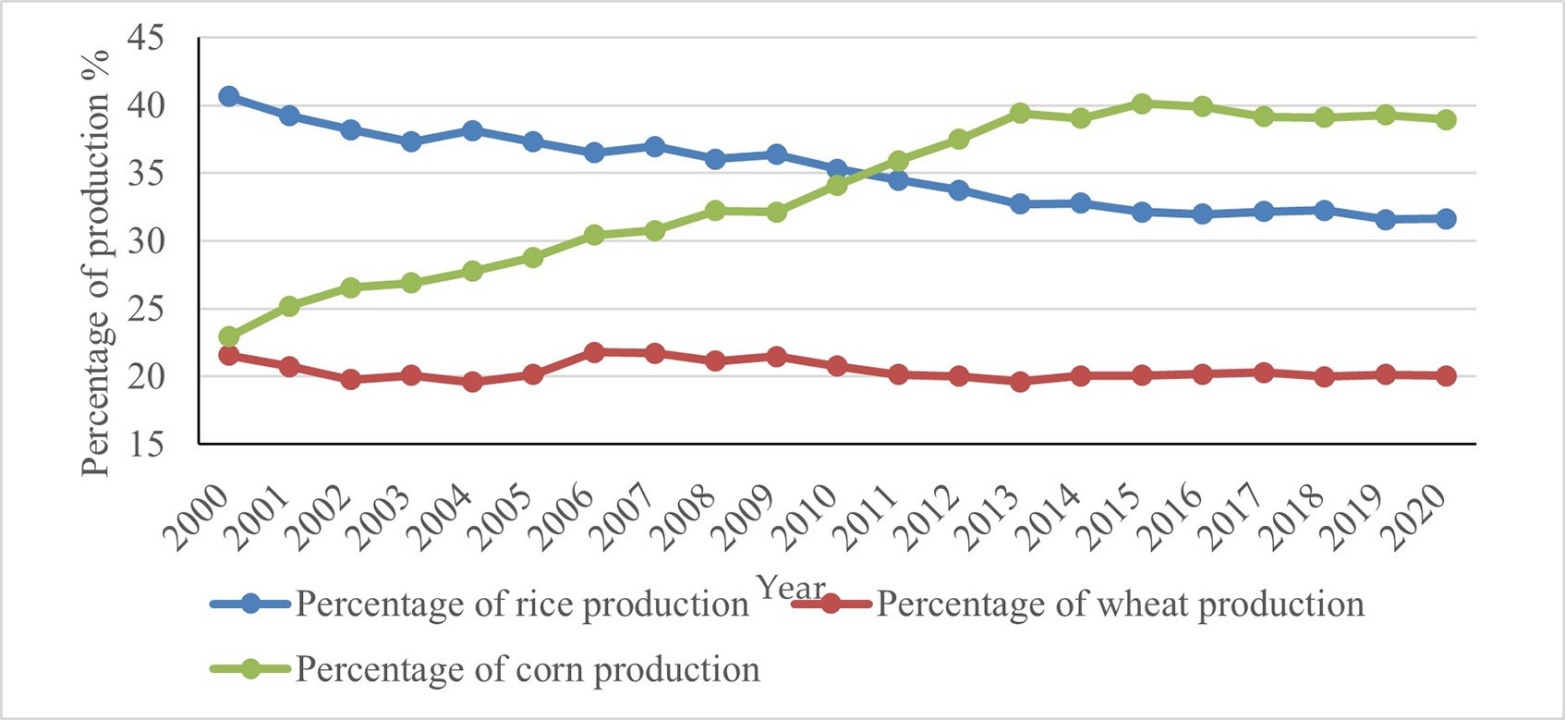
The proportion of rice, wheat, and corn production in China (2000–2020).

**Fig 4:**
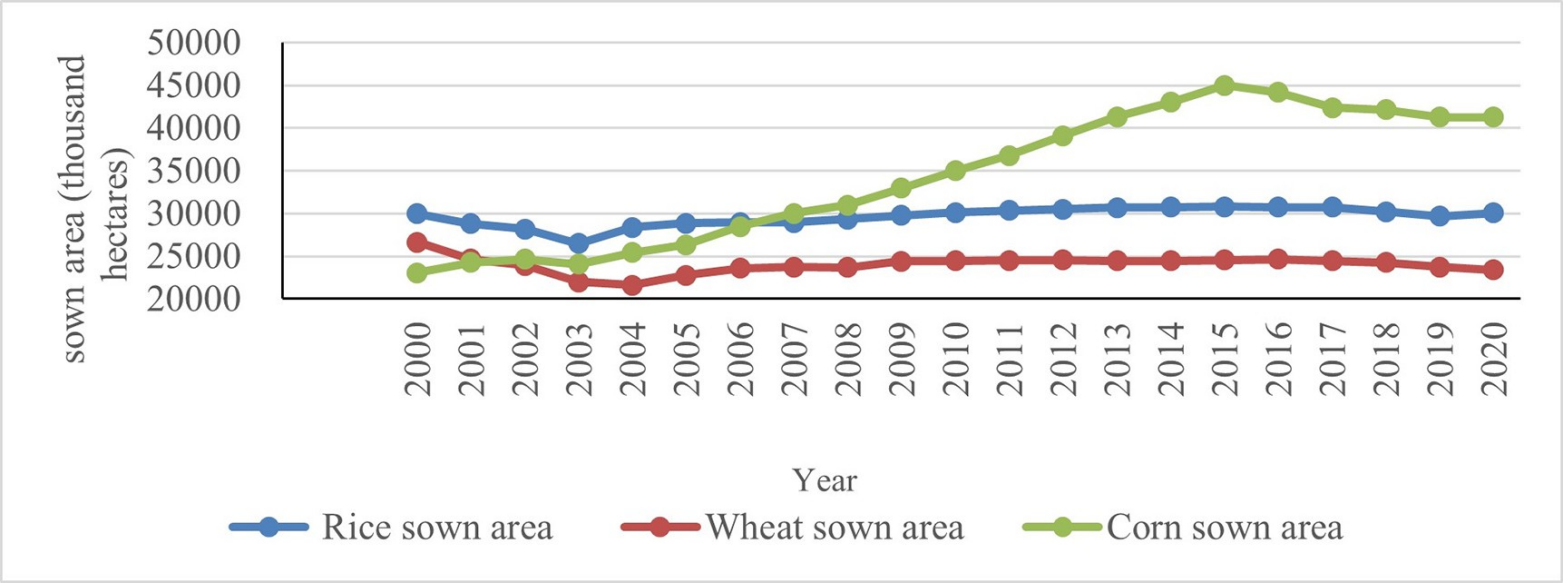
Sown area of rice, wheat, and corn in China (2000–2020).

(1) Changes in the yield structure of grain crops. From the year 2000 to 2020, there were significant changes in the yield structure of the three major grain crops: rice, corn, and wheat. Among them, the proportion of wheat yield remained unchanged at around 20%. The trend of changes in the proportion of rice and corn production is opposite, with rice production continuously decreasing from 40.66% in the year 2000 to 31.64% in the year 2020, and corn production continuously increasing from 22.93% in the year 2000 to 38.93% in the year 2020. Starting from the year 2011, the proportion of corn production began to exceed that of rice production, becoming China’s "first staple food" (Fig 3).

(2) Structural Changes in Sowing Area of Grain Crops. From the year 2000 to 2020, the trend of changes in the planting area of rice, wheat, and corn varies across the country. The planting area of corn has the largest change, reaching its maximum value (44968 thousand hectares) in 2015 and the minimum value in 2000 (23056.1 thousand hectares) during the research period. The former is 1.95 times higher than the latter. The year 2003 and 2015 are the dividing points of corn planting area, with corn planting area continuously increasing from 2003 to 2015 and then decreasing. During the research period, the sowing area of rice and wheat both decreased, and the trend of their changes is similar. Among them, the wheat planting area is divided into two stages based on 2004. From 2000 to 2004, the wheat planting area continued to decrease, reaching its lowest point (21626 thousand hectares) in 2004. After 2004, the wheat planting area increased, but after 2009, it stabilized and did not exceed the planting area in 2000. Similarly, 2003 was the boundary point for the planting area of rice. From 2000 to 2003, the planting area of rice continued to decrease, reaching its lowest point (26508 thousand hectares) in 2003. In the second stage, the planting area of rice increased after 2003, and in a few years after 2010, the planting area exceeded that of 2000 (Fig 4).

As for the three major grain crops, the increase in total corn yield is not only due to the increase in their planting area, but also closely related to the increase in their unit yield. On the other hand, rice and wheat are different. As the planting area of the two gradually stabilizes, the increase in their total yield is mainly determined by the increase in their unit yield. In terms of grain production structure, both the yield structure and sowing structure of grain crops show that corn has become the largest grain crop in China.

### Analysis of spatial characteristics of China’s grain production

#### Characteristics of changes in the spatial pattern of grain production

**Fig 5:**
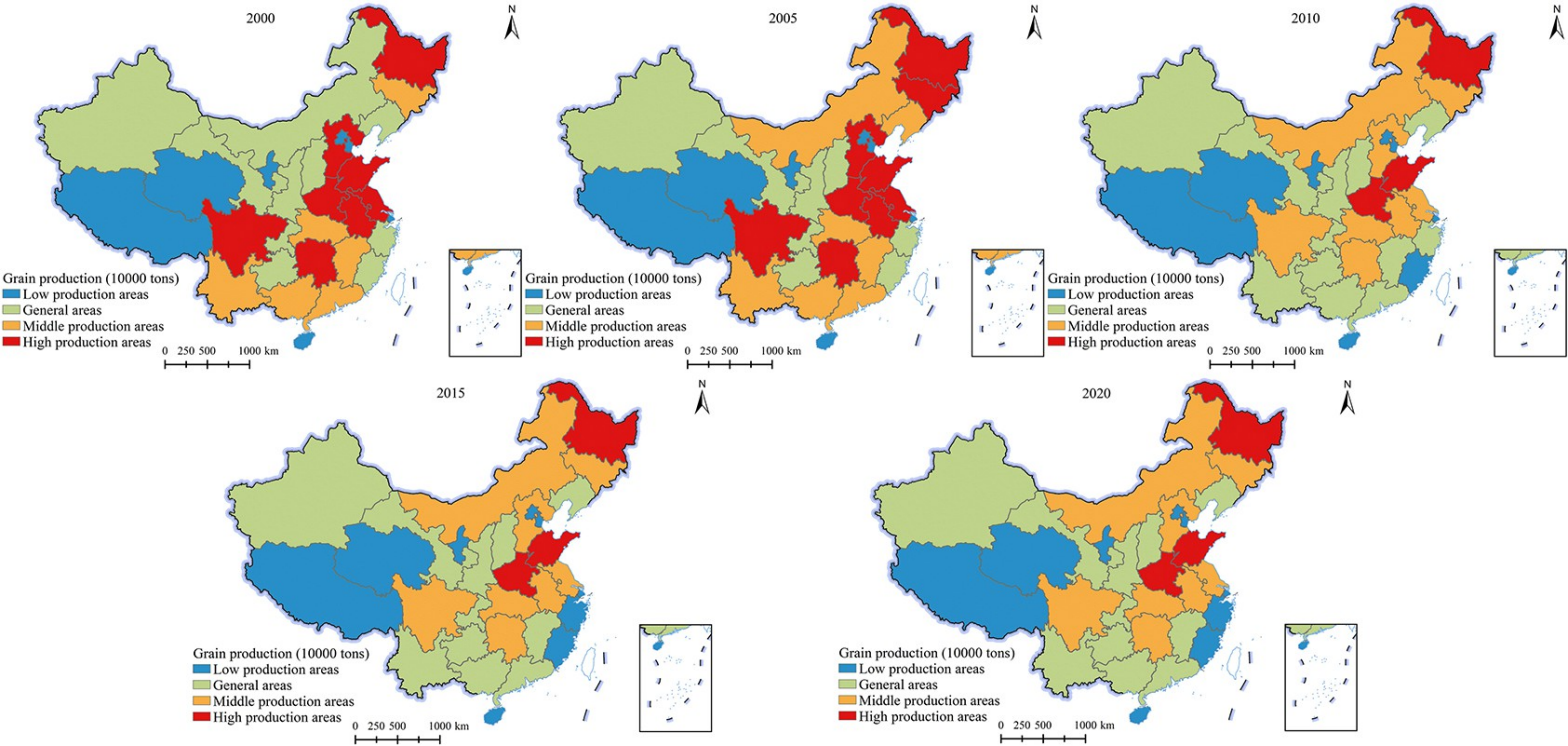
Changes in the spatiotemporal pattern of grain production in China (2000–2020) (GS (2019) 1823).

This study used the natural breakpoint method to divide the national provincial grain yield into low production areas, general areas, middle production areas, and high production areas (Fig 5). From the perspective of quantity changes, the provinces (cities, districts) in high production areas have experienced a slight increase followed by a sharp decrease and stable change, from 8 in 2000 (Heilongjiang, Hebei, Shandong, Henan, Jiangsu, Anhui, Sichuan, Hunan) to 3 in 2020 (Heilongjiang, Henan, Shandong).The number of provinces (cities, districts) in the middle class areas has shown a continuous increase and stability, increasing from 6 in 2000 (Jilin, Hubei, Jiangxi, Guangdong, Guangxi, Yunnan) to 8 in 2020 (Inner Mongolia, Jilin, Hebei, Jiangsu, Anhui, Hubei, Hunan, Sichuan).The number of provinces (cities, districts) in general districts has undergone a change of "decrease increase decrease stability", increasing from 10 in 2000 (Xinjiang, Gansu, Inner Mongolia, Liaoning, Shanxi, Shaanxi, Chongqing, Guizhou, Zhejiang, Fujian) to 11 in 2020 (Xinjiang, Gansu, Liaoning, Shanxi, Shaanxi, Chongqing, Guizhou, Yunnan, Guangdong, Guangxi, Jiangxi).The number of low production areas has increased from 7 in 2000 (Xizang, Qinghai, Ningxia, Beijing, Tianjin, Shanghai, Hainan) to 9 in 2020 (Xizang, Qinghai, Ningxia, Beijing, Tianjin, Shanghai, Zhejiang, Fujian, Hainan).

From the perspective of spatial changes, the spatial scope of high production areas has significantly narrowed, with Heilongjiang, Henan, and Shandong provinces becoming the main grain production areas. The distribution of middle class and general areas is relatively scattered. The distribution of low production areas is relatively stable.

#### Changes in the spatial agglomeration pattern of grain production

**Fig 6:**
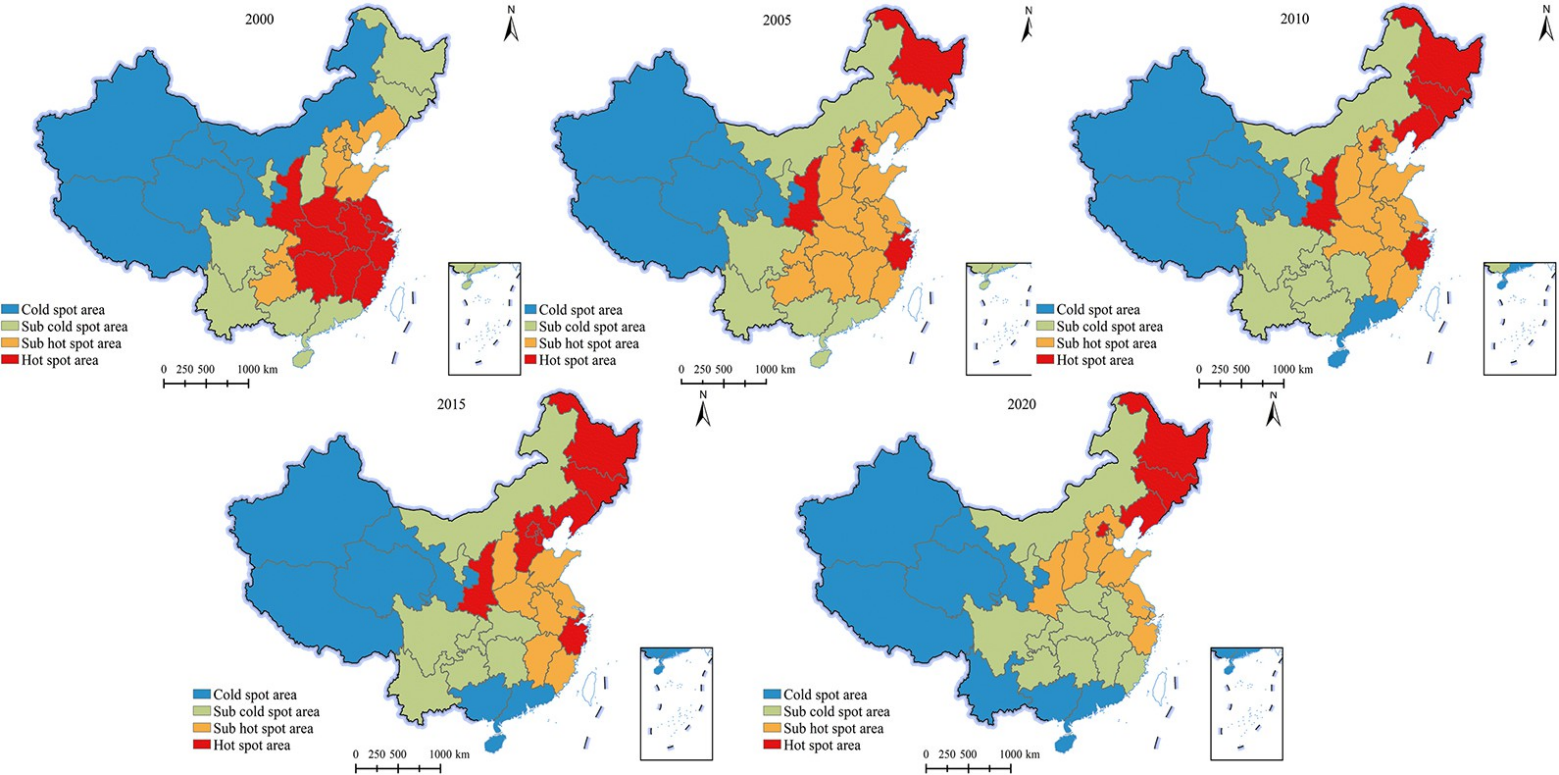
Evolution of the spatial agglomeration pattern of grain production in China (2000–2020) (GS (2019) 1823).

To analyze the changing characteristics of the spatial agglomeration pattern of national grain production, a local correlation index was used to divide it into cold spot areas, sub cold spot areas, sub hot spot areas, and hot spot areas using the natural breakpoint method from low to high. From Fig 6, it can be seen that many provinces have high (low) grain production areas that exhibit spatial agglomeration. From 2000 to 2005, there were significant changes in the spatial agglomeration pattern of national grain production, with the hot spot areas in the central and eastern provinces contracting significantly, while the cold and sub cold spot areas in the northeast contracted significantly. From 2005 to 2010, the sub hotspot areas in the central and eastern provinces significantly contracted, while the hot spot areas in the northeast region expanded significantly. From 2010 to 2015, the sub hot spot areas in the central and eastern provinces further contracted, with the northern provinces (Hebei) turning into hot spot area and the southern provinces (Hubei) turning into sub cold spot area. From 2015 to 2020, hot spot areas were concentrated in the Northeast region, sub hot spot areas were concentrated in the northern provinces, and most of the western and southern provinces were cold and sub cold spot areas. The focus of grain production shifted to the north.

#### Characteristics of changes in the distribution center of grain production

**Fig 7:**
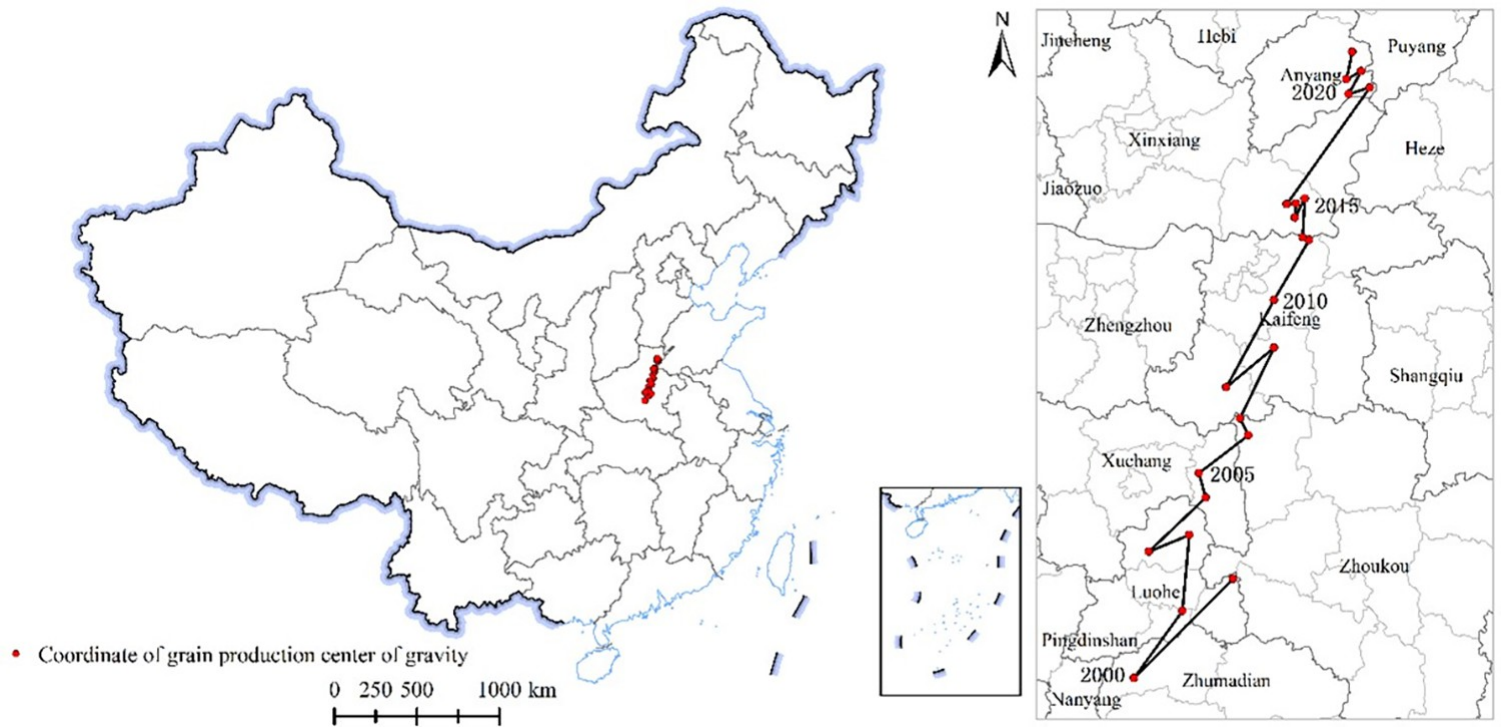
Migration trajectory of grain production center of gravity in China (2000–2020) (GS (2019) 1823).

To more clearly display the changes in the center of gravity of national grain production, the center of gravity migration model method is used to connect the coordinates of the center of gravity of national grain production in five years to obtain the trajectory of the center of gravity of national grain production (Fig 7). The geographical coordinates of the center of gravity of national grain production were calculated to be 113.77° -114.95° E, 33.34° -35.55° N, all located in the northern part of Henan Province. From Fig 7, it can also be seen that over time, the center of gravity of national grain production gradually shifts towards the northeast, indicating an uneven spatial distribution of grain production in China. Henan has become a key region for national grain production, and at five-time nodes, it has always been located in the high-yield area of grain production in China. This is mainly because Henan is located in a plain with a large arable land area, mainly planted with grain crops, suitable for large-scale production and mechanized operations. With the improvement of agricultural mechanization level and the promotion of high standard farmland construction, it has provided a solid foundation for the increase of grain production in Henan Province.

### Analysis of the driving forces of China’s grain production pattern

#### Factor detection analysis

**Fig 8:**
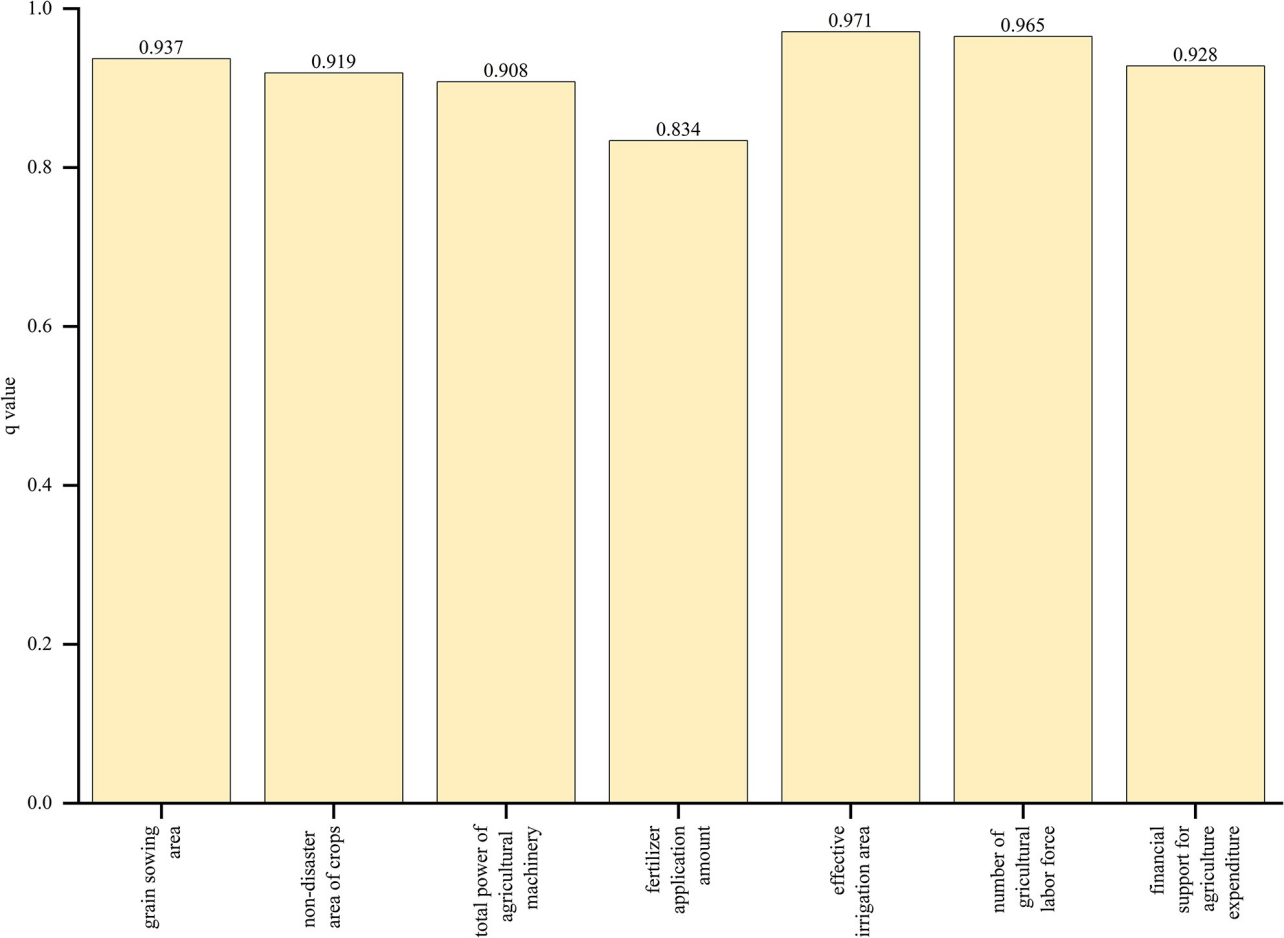
Factor detection results of factors affecting grain production in China (2000–2020).

Using a factor detector to detect the explanatory power q value of seven influencing factors on grain production, the higher the value, the greater the impact of this factor on grain production. The results are shown in Fig 8, and different influencing factors have different explanatory power on grain production. Among natural factors, the influence of grain sowing area is greater than that of non-disaster crops, while among capital factors, the influence of effective irrigation area is the greatest. Overall, effective irrigation area>number of agricultural labor force>grain sowing area>financial support for agriculture expenditure>non-disaster area of crops>total power of agricultural machinery>fertilizer application amount.

The q value of effective irrigation area is the highest (0.971) and significant at the 0.01 level, indicating that effective irrigation area is a decisive factor affecting grain production. Affected by natural geographical factors, the frequent occurrence of extreme weather in the northern region has had a significant negative impact on grain production. The increase in effective irrigation area helps to improve the ability of grain production to withstand extreme climate disasters, thereby ensuring the sustained and stable growth of grain production. As of the end of 2020, China’s effective irrigation area reached 69200000 hectares, accounting for 53.64% of the country’s arable land area, laying a solid foundation for the improvement of China’s grain production.

The q value of agricultural labor force ranks second (0.965), which is significant at the 0.01 level, indicating that the quantity of agricultural labor force is one of the important factors affecting grain production. Due to the fragmented scale of agricultural production and operation, the role of labor force in China’s grain production is more evident. In recent years, with the improvement of urbanization level, there has been a serious outflow of agricultural labor force, and some areas have experienced the abandonment of arable land, seriously affecting China’s grain production. In 2020, the number of employed people in China’s primary industry reached 177 million, accounting for 23.6% of the total employment in the three industries, indicating the gradual transfer of agricultural labor to the secondary and tertiary industries.

The q value of grain sowing area ranks third (0.937), which is significant at the 0.01 level, indicating that grain sowing area has a significant impact on grain production. Generally speaking, the sown area of grain is a direct factor affecting grain production, as an increase in sown area leads to an increase in grain production level. In 2020, China’s grain planting area reached 1.752 billion acres, accounting for 70.09% of the total crop planting area, laying the foundation for stabilizing grain production.

The fourth is the q value of financial support for agriculture expenditure (0.928), which is significant at the 0.01 level, indicating that financial support for agriculture expenditure plays an important role in changes in grain production. Policy factors are one of the important factors affecting grain production. The increase in financial support for agriculture expenditure can not only reduce the cost of grain production and directly promote it, but also convey the signal that the country attaches importance to grain production, thereby increasing the enthusiasm of business entities for grain production and having an indirect positive effect on changes in grain production. From 2000 to 2020, financial expenditure on supporting agriculture has been increasing year by year, which has played a promoting role in improving grain production.

The fifth is the q value of the non-disaster area of crops (0.919), which is significant at the 0.01 level, indicating that the non-disaster area of crops will still have a significant impact on grain production. The larger the disaster area of crops, the more grain production will be reduced, and vice versa. The disaster area of crops is greatly affected by natural factors, especially floods and droughts. If natural disasters occur in major grain producing areas, the adverse impact on grain production will be greater. In 2020, the disaster area of crops in China reached 7993000 hectares, a slight increase compared to 2019, but at a lower level between 2000 and 2020, indicating an improvement in China’s control over the impact of natural disasters.

The sixth is the q value of the total power of agricultural machinery (0.908), which is significant at the 0.01 level, indicating that the total power of agricultural machinery is also an important factor affecting grain production. With the improvement of urbanization level in China, the number of agricultural labor force is gradually decreasing, and capital replacing labor has become the development trend of agricultural production. The improvement of technological level makes the promotion and use of agricultural machinery possible. Similarly, in grain production, the improvement of mechanization level is inevitable, and thus the total power of agricultural machinery will have an increasing impact on grain yield. In 2022, the total power of agricultural machinery in China reached 1104 million kilowatts, playing an important role in promoting various aspects of agricultural production.

Finally, the q value of fertilizer application amount (0.834) is significant at the 0.01 level, and has a relatively small impact on grain production compared to other influencing factors. In fact, chemical fertilizers are one of the main input factors in the process of grain production. However, in recent years, in order to promote sustainable development of grain production and improve the quality of arable land, people often reduce the use of fertilizers and pesticides and increase grain production through green and environmentally friendly means. Moreover, in recent years, the total amount of fertilizer application in China has gradually stabilized, with the focus on improving the efficiency of use. According to statistics, the average application rate of fertilizers in China is twice the upper limit of safe application of fertilizers in developed countries, but the average utilization rate is only about 40% [22].

#### Interaction detection analysis

**Fig 9:**
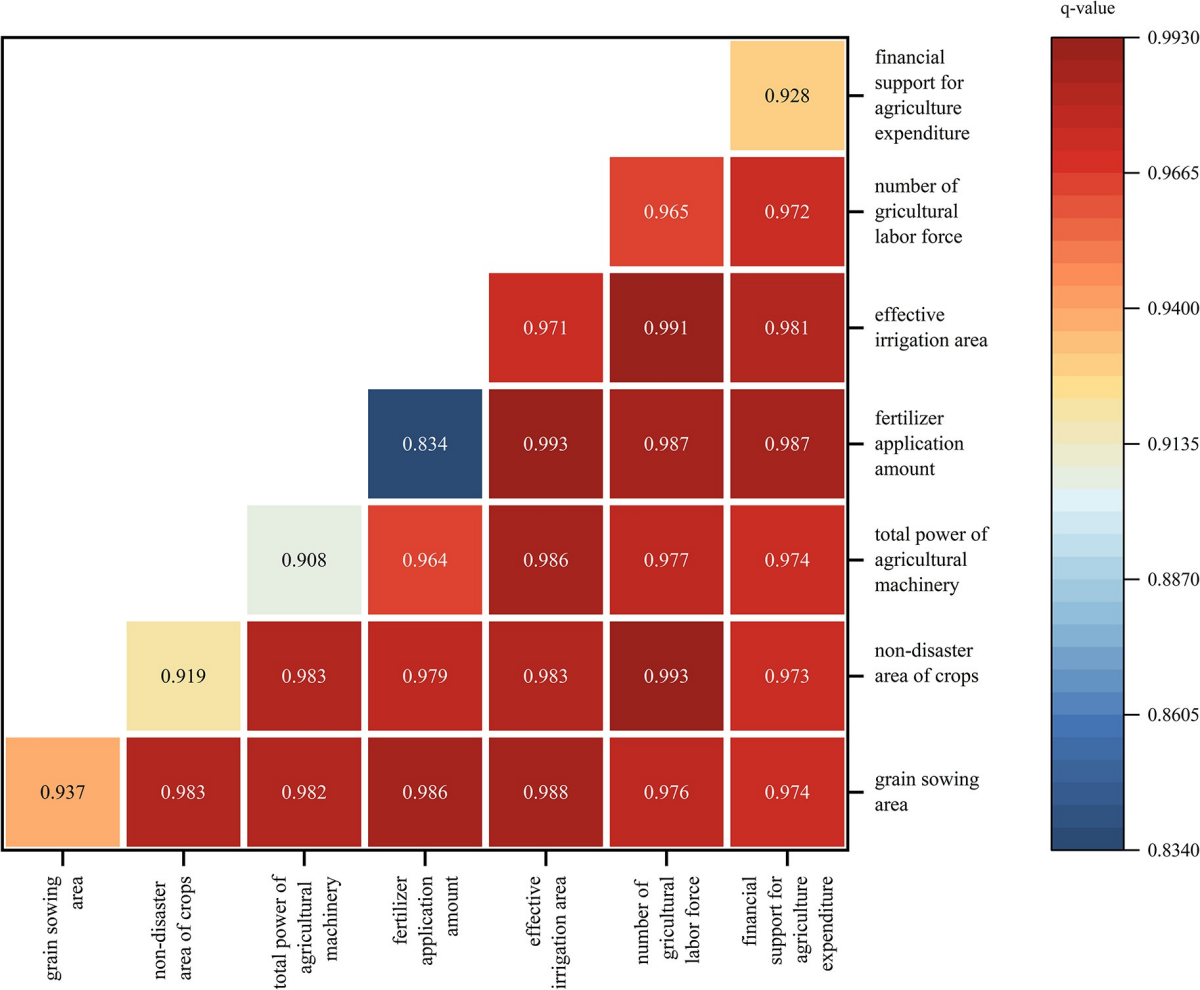
Detection results of the interaction between factors affecting grain production in China (2000–2020).

According to the results (Fig 9), the interaction between any two influencing factors has a greater impact on grain production than a single factor, indicating that the interaction between a single factor and any other factor is greater than its own individual effect, and all are enhanced by two factors. This also indicates that grain production is influenced by multiple factors and has complexity.

The interaction between influencing factors enhances the spatial differentiation of grain production. Among them, after the interaction with other factors, the impact of fertilizer application on grain production is significantly improved compared to when it acts alone. This indicates that in the current situation of low fertilizer utilization rate, the fertilizer application amount factor needs to rely on other factors to play a greater role, thus becoming an auxiliary factor in explaining grain production. Among the q values formed by all interactions, the interaction between the number of agricultural labor and the area of crops that have not been affected by natural disasters has the largest q value, indicating that the interaction between the number of agricultural workers and the absence of natural disasters has a significant impact on grain production. Moreover, the q value of the interaction between the two indicators in natural factors is relatively large, indicating that China’s grain yield currently requires a larger number of laborers under household production conditions, The dependence on natural conditions is still strong.

## Discussion

### Evolution of grain production pattern

This article found through research that from 2000 to 2020, China’s total grain production showed a trend of first decreasing and then increasing, that is, China’s grain production showed a "fluctuating rising" trend [23]. Among them, from 2004 to 2013, the overall national grain production showed an upward trend, with a phenomenon of "ten consecutive increases" in grain production [24]. The increase in corn yield has played a leading role in increasing the total grain yield [6].

In terms of the temporal characteristics of grain production, this article finds that the high-yield areas of grain production are mainly concentrated in the northern regions, especially in Henan, Shandong, and Heilongjiang provinces. However, in reality, there is a significant spatial imbalance in China’s grain production, with grain production gradually concentrating in the Northeast, Central, and East China regions. The overall spatial pattern of grain production shows an evolutionary trend of " retreat south and advance north " [25].

Another research conclusion of this article is that there is a spatial agglomeration feature in the same value regions of grain production, manifested as a clear positive spatial autocorrelation between grain production in various provinces and regions [17]. The grain production pattern has significant spatial spillover characteristics, and some provinces, regions and cities have "ripple effect" and "scale effect" in grain production spatial spillover [25]. The national grain production increase and decrease areas show a clear spatial agglomeration state, and the agglomeration state continues to strengthen. High and high agglomeration areas gradually shift towards the northeast agricultural area, while low and low agglomeration areas gradually shift towards the eastern and southern coastal areas [8].

In addition, this article found through research that the center of gravity of grain production is gradually shifting northward, which is consistent with the research of some scholars [26, 27], and further research also shows that the center of gravity of grain production in China is shifting towards the northeast, with most of the center of gravity located in the central region of Henan Province [17]. In addition, the production potential center of grain production space is also constantly moving towards the northeast, with a significant migration range [1].

From the above, it can be seen that the changes in China’s total grain production and production structure in the past 20 years are consistent with existing research. The conclusions of the grain production pattern also conform to the overall trend of "retreat from the south and advance from the north". The spatial agglomeration characteristics of regions with the same grain production value are consistent with the spatial autocorrelation and scale effects of grain production in existing research, The conclusion that the focus of grain production has shifted northward and concentrated in Henan Province is also supported by existing research.

### Driving mechanism of grain production

In terms of factors affecting grain production, sunshine, temperature, precipitation and natural disasters all affect grain production to a certain extent. Appropriate climatic conditions can improve grain production to a certain extent, while high temperature, rainstorm, hail and other natural disasters cause extremely serious damage to grain production [16]. This study found that effective irrigation area has the strongest explanatory power on grain production, in fact, the effective irrigation area has a significant positive impact on the growth of grain production in China [17], and is one of the main factors affecting regional changes in grain production [27], the impact of drought on grain production cannot be ignored [28]. The Non-disaster area of crops is an important factor affecting grain production, so the impact of natural disasters on grain production is significantly negative [14], and it still has a strong negative impact on grain production [18].

The sowing area of grain is one of the main factors driving the spatiotemporal changes in the main grain producing areas [19], and its proportion to the sowing area of crops has a significant positive impact on the growth of grain production in China [17]. The increase in sowing area plays an important role in the growth of grain production, and with the increase in sowing area, the unit yield of grain has also been improved [18]. However, maintaining the cultivated land area is necessary to ensure the sown area of grain [16]. The per capita cultivated land area has a significant positive impact on the total grain yield [17]. However, some studies have found that the contribution of cultivated land area to grain yield is a reduction effect. After all, in the context of accelerated urbanization and industrialization, it is expected to steadily increase the cultivated land area, it is basically unrealistic to transform the reduction effect of cultivated land area into a growth effect, thereby promoting grain yield [29].

In addition, the quantity of agricultural labor ranks second in terms of explanatory power for grain production. The quantity of agricultural labor is the main factor driving the spatiotemporal changes in the main grain producing areas [19], but there are also studies that have shown that labor input is not a driving factor for the increase in grain production [18], this may be related to the research area, such as the main production and sales areas, and different research methods may also affect the final results. The financial support for agriculture expenditure mainly reflects the important role of policies in grain production, and agricultural policies are the main factors affecting regional changes in grain production [27]. Government intervention and other factors will have a significant positive effect on the evolution of the spatial pattern of grain production [25]. The explanatory power of the total power of agricultural machinery on grain production is relatively small, but the mechanical input plays a significant positive role in the evolution of the spatial pattern of grain production [25], and the increase in mechanical input is an important reason for the increase in grain production [18], moreover, the total power of agricultural machinery has a significant spatial spillover effect on grain output [30]; The explanatory power of fertilizer application on grain production is minimal, and it also has a significant positive impact on the growth of grain production in China [17], but its impact shows a weakening trend [20]. In terms of the comprehensive effect of factors, this article believes that the interaction between factors can enhance the impact on grain production. Some studies have also found that the spatial spillover effect of effective irrigation area is significant, and the comprehensive effect of factors leads to the evolution of the pattern of grain production [17].

From the above, it can be seen that in the past 20 years, natural factors, policy factors, and capital factors have all had a positive impact on grain production in terms of the driving mechanism of grain production. The interaction effect of these influencing factors on grain production is also consistent with existing research. However, in terms of labor factors, the role of labor force on grain production is inconsistent with some research conclusions. This is related to the characteristics of China’s long-term large-scale small-scale farmers and the stage of industrial transformation. Due to the extremely complex nature of the grain production system, every step from sowing to production is easily influenced by various factors such as society, economy, and nature [31].

### Future research directions

This article takes the provincial level as the research unit, analyzes the spatiotemporal evolution characteristics of China’s grain production from 2000 to 2020 based on statistical data, and analyzes the factors that affect grain production. The aim is to clarify the pattern evolution of grain production and provide suggestions for improving grain production and ensuring grain security. However, due to limitations in data collection in some regions, it may have a certain impact on the accuracy of research results. Therefore, analyzing the evolution of grain production patterns on a larger regional scale and proposing countermeasures and suggestions to improve grain security levels are still issues that need further research in the future. In terms of factors that affect grain yield, the changes in natural conditions caused by global climate change and other factors may be a further issue that we need to pay attention to.

## Conclusions and policy implications

### Conclusions

Based on data from 31 provinces (cities, autonomous regions)(2000–2020) on grain yield, grain sowing area, main grain crop yield, and sowing area, combined with local correlation index, and center of gravity migration model, the spatiotemporal evolution characteristics of grain production during this period are analyzed, and the factors affecting grain production are analyzed. The conclusions are as follows.

1. From the year 2000 to 2020, the total grain production in China showed a trend of first decreasing and then increasing, while the unit area grain production in China showed an increasing trend. In terms of grain production structure, both the yield structure and sowing structure of grain crops show that corn has become the largest grain crop in China. Due to the influence of natural conditions and planting habits, more wheat and corn are planted in the northern region. With the improvement of people’s income level, the demand for high-quality food, especially high-quality beans and potatoes, is increasing. In the current situation where corn has become the "first staple food" in China, the food supply structure is difficult to meet the demand.
2. From the year 2000 to 2020, high-yield areas of grain production were concentrated in the northern region, and the same value areas of grain production showed spatial clustering characteristics, gradually shifting the focus of grain production towards the north. Henan has become a key region for national grain production. The severe grain shortage areas are mainly concentrated in the densely populated, low grain production, and economically developed southeastern coastal areas, making it inevitable to transport grain from the north to the south.
3. The results of the geographical detector show that China’s grain production is influenced by multiple factors, among which the effective irrigation area is the most important factor affecting grain production, followed by the number of agricultural labor force, and the third is the grain planting area. In addition, the impact of various influencing factors on grain production has increased after interaction, and all of them are enhanced by two factors.

### Policy implications

To optimize the layout of China’s grain production and improve the level of food security, the following aspects should be taken into consideration.

Firstly, stabilize grain production and reduce the pressure on grain inventory. Affected by external factors such as the global epidemic and the Russia-Ukraine conflict, it is urgent to work together from supply, market, demand and other aspects to continuously enhance the stability of grain production. On the supply side, actively cultivate new agricultural management entities such as family farms and farmers’ cooperatives to become the main entities of grain production. Establish a sound mechanism for the formation of grain prices, fully leverage the leading role of the market in the formation of grain prices, monitor grain market prices and carry out grain storage work, and gradually improve the phenomenon of inverted grain prices at home and abroad. Guide grain consuming enterprises to carry out technological innovation to increase the added value of grain, improve their ability to control fluctuations in grain prices, fully leverage the important role of grain consumption in grain destocking, and form a virtuous cycle of grain production, storage, and sales.

Secondly, implement regional grain security responsibilities and improve the efficiency of grain circulation. Clearly delineate the grain security responsibilities of each province (district, city), enhance the grain production capacity of major grain producing provinces, Stabilize the level of grain production in provinces (districts, cities) with equal production and sales, as well as in the main selling provinces (districts, cities), and increase compensation for major grain producing provinces on the basis of completing the national grain production target tasks. Establish a long-term mechanism for compensating for grain security responsibilities and benefits between major grain producing provinces (districts, cities) and major grain selling provinces (districts, cities). At the same time, we will accelerate the construction of grain reserve material platforms in various provinces, cultivate moderately sized grain trading markets based on regional characteristics, encourage grain circulation enterprises, farmer cooperatives, leading agricultural industrialization enterprises to become the main body of grain circulation, accelerate the transformation and upgrading of storage facilities in high-yield provinces (regions, cities), reasonably layout grain circulation infrastructure, and form a grain circulation network that closely integrates and effectively connects railway, highway, and waterway transportation.

Thirdly, promote the construction of high-quality grain projects and adjust the structure of grain production. Guided by the "big food concept" in grain production, we will launch high-quality grain construction projects in the main grain producing areas of the north, ensuring the safety of main grains while achieving diversified development of grains and optimizing supply structure. With the improvement of people’s income level and the further upgrading of consumption structure, the indirect consumption of grain will also increase. It is necessary to appropriately increase the planting area of feed grain, and at the same time, reasonably control the proportion of industrial grain to avoid further exacerbating the contradiction between industrial grain and human and livestock grain.

Fourthly, we must strictly implement the responsibility of protecting arable land and scientifically apply production materials such as pesticides and fertilizers. Local government departments should fully implement the responsibility of protecting arable land, provide special protection for permanent basic farmland, implement dynamic management of arable land divination and balance, and complete the national target task of arable land area. At the same time, seize the opportunity to renovate rural empty and abandoned homesteads, and use the urban-rural increase and decrease linkage policy to promote the efficient use of rural land resources. We will firmly uphold the red line of 1.8 billion acres of arable land, stabilize the grain planting area at 1.76 billion acres, curb the "non-agricultural" trend of arable land, prevent the "non-grain" trend of arable land, increase the construction of high standard farmland, transform medium and low yield farmland, improve the comprehensive production capacity of China’s arable land resources, and achieve the goal of "storing grain in the land". In addition, the scientific use of fertilizers is an important way to achieve stable grain production, increase production, improve grain quality, and ensure grain security. In the process of grain production, the use of organic fertilizers should be strengthened to improve the utilization efficiency of fertilizers and pesticides, while reducing the proportion of fertilizers and pesticides used.

Fifth, attach importance to the construction of agricultural infrastructure and stabilize the number of agricultural laborers. Increase investment in farmland water conservancy facilities, improve the farmland water conservancy irrigation system, and increase the effective irrigation area of farmland. At the same time, establish a disaster prevention, warning and control mechanism for grain production, reduce the impact of drought and other disasters, and improve the ability of farmland to resist natural disasters. Against the backdrop of increasing urbanization levels, stabilizing the number of agricultural laborers has a significant impact on the increase in grain production. In addition, agricultural machinery plays an important role in grain production, and improving the level of mechanized farming is an important means to ensure the improvement of grain production. Government departments should increase fiscal expenditure on supporting agriculture, increase policy tilt and financial support, increase the level of grain subsidies and agricultural machinery subsidies, increase the enthusiasm of main grain farmers, expand grain planting area, and lay a solid foundation for the improvement of grain production.
